# To know or not to know? Not the only question in familial breast cancer risk communication

**DOI:** 10.3332/ecancer.2011.239

**Published:** 2011-12-13

**Authors:** C Maddock, D Schrijvers, MRD Turco, L Marotti, R Sullivan

**Affiliations:** 1 On Behalf of TENOVUS, Cardiff, UK; 2 European Cancer Organisation (ECCO) Brussels, Belgium; 3 EUSOMA, Firenze, Italy; 4 Kings Health Partners Integrated Cancer Centre, Section of Oncology, Guy's Hospital, UK

## Abstract

**Background:**

Breast cancer is the most frequently diagnosed cancer and the leading cause of cancer death in females, 5–10% of these breast cancers occur in women because of an inherited mutation. The term ‘risk’ in relation to familial cancer can have multiple meanings for both clinicians and patients. Failing to identify and address this may impair effective communication and informed decision making and adversely affect the quality of patient care.

The aim of this research for the Eurocancercoms project was to explore patients' experience of risk communication in breast cancer and to investigate a mechanism for sharing these experiences using a filmed round-table discussion (RTD).

**Methods:**

A filmed RTD with six women who had experience of, or some connection with familial breast cancer was conducted. Criteria for inclusion included a willingness and ability to participate in the discussion in English and to be prepared for the discussion to be hosted online with opportunities for others to view and comment.

**Results:**

The main findings are presented as key themes and issues arising from the RTD. There was consistency in the group on the need for improvements to the risk communication process as a whole and in particular around onward diffusion of information i.e. ‘Telling the family’. There were differences regarding ‘wanting to know’ their genetic status.

**Conclusions:**

The perception of cancer risk in the narratives stems not only from the way risks are stated, but from family history, personal experiences, cultural norms and beliefs and therefore a multifaceted approach to risk communication addressing these issues is necessary to ensure the patient fully understands the potential risks. There is a balance when attending to patient's information needs, as to what level and amount of information is required by the individual at a particular time and communicators need to be able to tailor information accordingly.

## Background

Breast cancer is the most frequently diagnosed cancer and the leading cause of cancer death in females [1], 5–10% of these breast cancers occur in women because of an inherited mutation. According to estimates of lifetime risk, about 12% of women in the general population will develop breast cancer sometime during their lives compared with about 60% of women who have inherited a harmful mutation in BRCA1 or BRCA2 (in other words a woman with inherited BRCA1/2 mutation is about five times more likely to develop breast cancer [2]).

Genetic testing can now establish if a person carries a mutation in the BRCA gene and hence is at increased risk of breast cancer. The term ‘risk’ in relation to familial cancer can have multiple meanings for both clinicians and patients. Failing to identify and address this may impair effective communication and informed decision making and adversely affect the quality of patient care. Similarly, lack of efficient communication among the wider European cancer community about familial cancer, including within patient communities, remains a significant barrier to access quality information and collaborative approaches to improvements in best practice. When these issues are resolved health professionals can work more effectively with patients and can therefore enhance the quality of their care through patient and professional involvement in decision making.

The aim of this study was to focus primarily on patient's perspectives of the current methods and existing problems in communicating risk in familial cancer in their locale and to explore mechanisms for sharing these experiences. We wanted to enhance understanding of risk communication and effective strategies to enable its optimum use and benefits whilst minimizing potential harm associated with lack of, or poor, communication.

Risk communication concerns the sharing and discussion of information about the harms and benefits of different options in health and health care [3].

## Methods

This research was conducted on behalf of the Eurocancercoms project which was funded by an FP7 European Commission funded project, grant no. 230548. The study was approved by the independent Eurocancercoms Ethics Committee in February 2010.

### Recruitment to round table discussion (RTD) and post RTD review

Recruitment followed a pragmatic approach as we planned to hold a filmed RTD with women who had experience of, or some connection with familial breast cancer. We wanted to obtain, in particular, a European patients' perspective of risk communication and to learn about and share best practice from diverse services in several countries. It was also considered useful to have contributions from health professionals with expertise in the fields of medical genetics and cancer communication. Criteria for inclusion included a willingness and ability to participate in the filmed discussion in English and to be prepared for the discussion to be hosted online with opportunities for others to view and comment on the discussion. Europa Donna (the European Breast Cancer Coalition) was the main contact for proposing participants for the RTD who would be comfortable discussing their own experiences of risk communication in breast cancer and those of others they had come into contact with via this connection or via other local advocacy groups.

The European Breast Cancer Conference held at Barcelona from the 24–27 March 2010 was an ideal opportunity and situation to bring together the most appropriate people within the proposed time frame. The discussion did not form part of the conference, but was held at the same venue.

All participants were given a copy of proposed questions for inclusion in the RTD at least 2 weeks before the RTD and were given the opportunity to select questions that they were comfortable answering. They also had the opportunity to make further suggestions for discussion topics. All women agreed in advance that they were happy to answer the proposed questions. One participant responded to the questions in writing pre and post RTD (comments outside the RTD are identified as such in any text quoted).

Additional information and consent forms were sent to the participants. The duration of the filming and preparation was estimated to be about 2–2^1/2^ hours. It was clarified that the women would be able to review the completed film and ask for any sections that they were not happy with to be removed.

In order to test the concept of making the RTD available online to serve a wider audience, we contacted Macmillan Cancer Voices who put us in contact with individuals with an interest in BRCA breast cancer to review and comment on the RTD. The future aim was to provide this online discussion as a resource that could offer a source of information and give the opportunity to share thoughts, experiences and recommendations to a wider audience.

### Round table discussion

A focused discussion or focus group can offer a broad overview of issues and a range of perspectives and experiences of the group which can then be reviewed on a number of levels: for example on areas of consensus, disagreement and clarification [4].

A RTD was selected for this research to allow us to consider the participants views at an individual and group level, with an equal voice to all and also to continue this ethos through into an online discussion following its filming. The RTD followed a semi-structured format following the pre-agreed questions but allowing divergences due to the group interaction.

The question topics were chosen as a result of a review of the subject area and guided by Edwards et al's, 2006 document, Effective Risk Communication In Clinical Genetics: a systematic review [3].

### Analysis

The RTD was transcribed verbatim and data were analyzed thematically. A theme is a pattern found in the information that can describe and organize possible observations and can lead on to interpretation of aspects of the phenomenon [5]. Themes were generated inductively from the raw information but were informed by previous research.

Participants were encouraged to review the filmed discussion and contribute any additional clarifications or remarks. Comments made by two other BRCA affected women (recruited from Macmillan Cancer Voices) who reviewed the video clips have also been used to complete the data set along with the transcribed RTD (these are identified later as R7 and R8).

## Results

Six women took part in the RTD. The patient's representatives were from, Spain, Denmark, Ireland, and Holland. Two of the women were BRCA carriers and all had links with advocacy organizations in their countries. Invited experts were a professor of psycho-oncology and a senior research fellow in medical genetics. The RTD was filmed and edited into smaller sections that were hosted on the Eurocancercoms and ecancermedicalscience websites following approval of all participants.

## Main findings

The main findings are presented as the key themes and issues arising from the RTD. There was consistency in the group around the need for improvements to the risk communication process, particularly around information and its onward diffusion i.e. ‘Telling the family’ and differences regarding ‘wanting to know’ their genetic status. This was evident from the responses and the more extensive discussion on these topics.

The women shared their perspectives and experiences of breast cancer or risk communication and those of others in similar situations from their personal or advocacy group connections. The additional health care professional perspective contributed examples of research and their knowledge of services relevant to the discussion. The richness of the data emerges from the group dynamic and their diversity as not everyone had the same views and experiences due possibly to differences in, for example age, education, and in particular in this situation, across countries.

The following themes were identified from the RTD. Key quotations are included in the text to illustrate the theme and to show that within the topic there exists a range of comments, sometimes opposing.

The participants are identified as HCP1, HCP2 (health care professionals), P3-6 (patient representatives) where P4 added comments pre or post interview = 4a and 4b, R7, R8 (Macmillan Cancer Voice reviewers).

### Cancer genetic services

The RTD was introduced with a brief overview of cancer genetic services so this not only set the scene but also provoked some discussion about current service provision and recommendations for potential improvements. A person entering the cancer genetic service was described as *‘there for life’* due to the changing information and support needs at *‘different stages through the journey’. HCP2.*

There were several positive comments about the service, being *‘non directive’* and allowing ‘*sufficient time’* to discuss things, but disappointment with lack of support when passing on the news to family members:

‘*Twelve years ago you just got a letter with your diagnosis and to tell your family about it and I’ve heard from other people it’s still the same*' P4.

Involving patients in service redesign was advocated including involvement in guidelines.

‘*How is the whole process of communication experienced by people who have a predisposition, what do they need, what have they missed and what solutions are proposed*’ P4a.

“*persuading, …professionals not just to listen to women but actually to convene groups of people and to try and be informed…. looking at how populations get information and how they interpret general information…if that were pursued with more vigor certainly in Ireland I think we’d have better outcomes”* P5

There was recognition that a key beneficial difference with specific cancer genetic services compared to consultations with general practitioners (GP) was the planned time for discussion.

‘*not like your six minutes with your GP in the UK*’ HCP2.

### Information and support needs

The information and support needs category had several subthemes. There were comments from most of the women about the amount and from some the complexity of information making it difficult to understand.

### Amount and format of information

*‘for some people just the fact that there’s a lot of information or that some of it is difficult to understand … there's a great deal to be done still… about simplifying information, about making it very clear, about staging it properly, about explaining language, about all sorts of very basic stuff*' P5

Two of the women mentioned that information should be given in a variety of formats such as written information, DVDs and recommendations for internet sites to aid understanding: the formats reflecting diverse preferences, perhaps depending on their generation.

*the younger women in that group who are mutation carriers are…. increasingly IT literate and will need that information online … not boring patient leaflets that we've been doing for years* HCP2.

*but I would prefer a booklet or leaflet dealing with it. Booklets can be distributed amongst family members too. Information written sensitively and well can be picked up and read at leisure and then read again. Whereas when someone is talking to you, telling you carry a rogue gene, you are not taking it all in. When you have calmed down, reading information will be much better* R7.

### Emotional responses to information

There was recognition that the amount and complexity as well as the ramifications of the information divulged to them could contribute to confusion resulting in negative emotional and psychological effects.

*because people don't understand it fully, have different reactions and estimations of- and fear for their own risks* P4a.

*I did find my first appointment traumatizing. There was both a consultant and a counselor present, but they did nothing to alleviate the grief I was feeling after all the information I was given… I had no idea what I was expecting at the appointment, and then to be informed that I was a very high risk of a recurrence and should consider having both breasts removed and have reconstruction was too much to take in. I left the appointment very confused and not sure what to do* R8.

*if you get a DNA test result, it's like you get to hear you have cancer and giving a lot of information at that time doesn't work, because you are so stunned, …that you don't even hear the information… the real information, has to come in time*' P4.

### Staging and sources of information

One of the HCP participating agreed as *‘assimilating the implications of that, the consequences of taking different sorts of actions, can only be seen as a process’* but was concerned about the possibility of people looking around frantically on their own in a state of panic trying to fill gaps in their knowledge so advised:

‘*give people information knowing that they won’t take it on board, lots of written information, access to the sites, the trusted sites… because… there's a mass of really bad misinformation.. giving them a clear structure, a time frame when they can come back and discuss*' HCP1.

There was endorsement during and post RTD, for the role of advocacy organizations or individuals who could offer information and support in these circumstances.

*We organize a conference on hereditary aspects of breast and ovarian cancer every two or three years: …. with lectures and workshops for 250 or 300 people, all members from families with a predisposition, or possible predisposition. We give them information about all kind of subjects related to their predisposition and the choices they have to make. The lecture on breast reconstruction for example is always a topic attended by about 100 women and their partners. And the question part at the end of all sessions is always very important. Also to health care professionals, because they can hear what is in the people's mind after a period of time* P4b.

But some concerns were expressed by HCP1

But I do worry a little bit about the ‘be like me’, …about leaving it all to advocacy groups as well because you're going to be talking about you, your experience. She went on to emphasize that women can react in ‘utterly different ways

The role of the media in information dissemination was recognized both as a potentially useful source for raising awareness but also confounding by sending out mixed messages.

*The messages …whether through a press release from a respectable organization or by whatever means are actually still quite mixed. On the one hand you're being told, here's a great new treatment… and survival rates are going up and alternatively and in fact at the same time, you're being told, we need more money for research to beat this killer disease and the two things are going on side by side so which one do you listen to?* P5

One of the women had discovered about the possibility of having a genetic test for breast cancer in 1988 (not widely available at this time) via a newspaper, the other BRCA carrier through a ‘throw away remark’ in conversation.

### Communication and communication needs

The exchange of information is fundamental to cancer genetic counselling [6] and the giving and receiving of information as well as the specific skills of the HCP as communicators were discussed at some length alongside the recognition that *‘we know that people are very affected by the way in which options and risks are actually framed’* HCP1.

### Skills of communicators

‘*so much of this,…depends on the skill of the people who are giving the information and the nuances that they’re able to bring to that in reading whoever it is who's sitting in front of them*' P5.

*First recognize how the individual reaction could be: is it a person who wants to know all about its own medical situation or are they better off by keeping it out of their emotional system. How does each individual cope with difficult information about diseases in general. Do you want to hide, or do you seek information* P4a.

It was suggested that HCPs ‘*familiarity (with topic and language) prevents people from understanding what the person in the room is hearing*’ P5. This resonated with one of the HCPs:

‘*I think you’re absolutely right that people who are in the health services, … become inured to the language they use and forget that the rest of the world haven't got a clue what they're talking about half the time*'

There was some consensus within the group that speaking to others in similar circumstances could prove beneficial, and this was also supported by one of the reviewers.

‘*I think it’s definitely good to speak to someone in a similar situation'* R8

*… is one of the most important things of all, to know that there are other people out there who've been through this before you and can perhaps help you* P6.

### Techniques and tools

There were suggestions that there needed to be different ways of getting across the key genetic risk information, particularly on an individual level, but also to raise general public awareness.

‘*The people who are going to help their patients most are those who can see quite quickly that the person really isn’t with them, and has got a vast repertoire of different ways of describing things…, we have got so many wonderful ways now… with graphics and cartoon, to find different means to help people understand in ways that are appropriate for them* HCP1.

And to emphasize that one technique will not work for all:

‘*being shown graphics and cartoons to help understand would not be what I would want*’ R7.

One of the HCPs described a population approach in America to raising awareness of genetic risk on a wider scale and described ‘*the Turkey Talk, where you should sit down with your family and talk about your family history and what’s running through your family. I think the Surgeon General in the States is promoting this as, let's all increase awareness about family history'* HCP2.

A more rigorous approach to identifying specific patient information requirements was recommended by HCP1, possibly including more detailed patient profiling initially to determine what approaches to risk communication would be most appropriate to the individual.

*it's a really, really difficult thing to predict how somebody's going to behave unless you, in your screening, do all these other sort of tests as well, then you can help advise people a little more carefully* HCP1,

### Interpreting risk

Another important issue raised was that of interpreting risk and the acknowledgment that for various reasons including understanding of basic health information (health literacy), personalities and pre-existing beliefs and taking into account the risk communicator's ability to convey information, there was a distinct possibility that different women would interpret the same information in different ways.

‘*I just think that you’ve got to get on with life, there's worse things that can happen, you know, I actually feel quite a lucky sort of person'. So people reinterpret those risks according to their own personal sort of values, experiences, predispositions* HCP1 (describing type of comments heard during her research).

‘*the real issues here are not what the objective truth might be but about who’s doing the telling, how they're doing the telling, how it's being received, what people are taking home with them, how that's being processed*' P5.

There was also a stark reminder for health professionals that ‘*merely informing somebody of their risk, so that they could repeat it back to you, is no predictor at all about how they will then interpret it and the actions that they’ll then take*' HCP1.

### Options and decision making

Women (particularly younger women) who carry a BRCA mutation may face difficult decisions regarding risk reducing strategies such as surveillance or prophylactic treatment options and these choices may have short and/or long-term consequences and significant impacts on their breast cancer risk. There can be negative impacts regardless of the choice. Information was seen as a resource to enable people to make decisions.

*The options are, to go away and do nothing, which some will actually do and just be enormously anxious or perhaps to have bilateral prophylactic mastectomy although they might still be at risk* HCP1.

*If you know, you are warned that you are at high risk and you can get surveillance so that gives you some security and you can take your preventative options like surgery or whatever* P4.

*And what happens when my daughters are positive and they will never have a cancer, you know, I don't want them to make surgery and prevention because they're young, they want to have more children, they want to give the breast, you know, they want to feed their children…* P3.

There was some discussion about the ‘meaning of breasts’

*I think all of those social meanings of what a breast is, come into it and all of the stuff about sexualisation of women's lives and all the rest of it but it's… So I think that there's something very specific about this* P5

Whatever symbolism and its meaning surrounds women's breasts; the thought of or action of their removal can cause enormous distress.

*What I think affects you when told you either have breast cancer or have the gene which makes you susceptible to breast cancer, is your actual breasts! For younger women and older women too, don't forget that, the loss of a breast is awful to contemplate* R7.

The reviewer goes on to say that ‘breast reconstruction is excellent and that not looking good should be the last of their worries. *It is thought by doctors – men especially – that women over 55 do not need their breasts. This is the NHS point of view. The private sector thinks differently*.’

This perspective, not necessarily representative of everybody's experience of the NHS, sadly may resonate with a number of women. It indicates not only the devastation caused to some women by the prospect of loss of their breasts but also the added distress that those involved in their care do not fully consider the significance and emotional impact that this may have on them.

### Wanting to know about genetic status or not

There were different reasons cited for wanting or not wanting to know their genetic status. One participant was concerned about her daughters, but was reluctant to have the test because of the perceived ramifications for the daughters and decisions they may have to make regarding prophylaxis.

*I just won't like to know if I’ve got a genetic cancer because I’m worrying about my daughters of course but what should they do now* P3.

For the two women who had confirmed BRCA mutation, the decision to get tested was knowledge that could inform choices which could affect their health care decisions and support ongoing plans for themselves or others. For one the imperative was *‘needing to know in order to be able to take whatever action was necessary and to be able to plan because the person who looks after me is me so I needed the information’* P5.

For the other BRCA +ve woman knowing her genetic status was a responsibility as she wanted to ensure her daughters had the information they needed to be able to take preventative actions. This concurs with Foster *et al*'s research indicating that responsibility to others serves as a primary motivation for undergoing BRCA1/2 testing [7].

*I'd like to know if my breast cancer is hereditary because I have two daughters and I’d like to know for them* P4.

Another reason not discussed in the RTD but identified by one of participants and a reviewer post RTD was that of the problems associated with getting medical insurance and that this may indeed impact on individual's decisions to get tested

*Please don't forget the insurance risks. If a family member wants to ensure her or his life, they do not want a genetics test to tell them that they are predisposed towards getting a certain killer disease. This alone would prohibit an awful lot of people from getting themselves tested. And I don't blame them either* R7.

### Telling the family

This responsibility to others included the extended family and the following comment expresses this.

‘*I felt it a sort of obligation to tell my family because knowing is a responsibility but suppose if some of my cousins have breast cancer and I didn’t tell them. So I thought I have to tell them and what they do with it, sorry, but that is… What each person is deciding*… P4

This led onto a much discussed issue around ‘Telling the family’ and the ethical, emotional and practical considerations involved in that. There was recognition that telling the family was not an easy task and that people were concerned with their ability to pass on complex and potentially distressing information when frequently they may only recently have had their own genetic status confirmed.

*people always have difficulty in when and how to tell their children* P4a.

*There is absolutely no guidance in how to inform your family members. Perhaps that ‘s why it is often received negatively in your family because people don’t understand it fully* P4a.

Also there was the issue of relationships within families which are not always simple.

‘*but relationships in the family are diabolical… I had to write a very peculiar letter …I’ve been diagnosed with this, your daughter may well be at risk*' P5

Further comments by one of the participants and reviewer added to views, not only on difficult family situations but also the moral aspect of imposing your views and sense of obligation on to others.

‘*Family feuds are often at hand, apparently caused by not getting any support in how to tell your family, how to tell your children and how to cope with genetic predisposition …*’ P4b.

‘*Families are difficult at the best of times and dropping a bomb shell like this on assorted cousins and aunts is not a good idea and would not work. It is entirely up to them. Trying to tell extended family that they should be genetically tested is a mine field*’ R7.

Appreciation of the difficulties of and the skills required by HCP to ensure effective communication that will enable people to make informed choices at the same time considering *‘underlying beliefs’* led to one woman questioning the wisdom of expecting recently diagnosed women to inform their family of their genetic status thus adding an unnecessary ‘burden’ on them.

‘*The idea that you leave it to the individual to do it, then gives them a sort of burden of conscience, which seems to me to be ridiculous on top of everything else. And also… And a sort of abrogation of responsibility, I suppose, in the same sort of way, I think, I have to say I find it very strange and I don’t know whose interests it's intended to serve*' P5.

Several of the women agreed there was a need for improvements to current practice.

‘*would it not be a good idea to get the genetic counselor involved and explain through him/her the risks involved rather than leaving it to the individual concerned?*’ R8.

### Service delivery in other countries

One of the objectives of the RTD was to ascertain if there were models of good practice that could be shared across borders. Indeed the women did bring to the discussion some examples of what they considered to work well or could be improved based on their own knowledge or experience.

*(re. Denmark): we have these marvelous cancer records* P6

‘*There’s a blog on the internet where people can write in and I understand that they get up to between six and seven hundred letters every month from people who have problems with breast cancer and particularly with genetic breast cancer* P6.

These particular services were well thought of by both reviewers causing one (R8) to comment ‘*I must say the service in Denmark seems excellent.’ and the other to ‘wish this country would adopt the records method of Denmark’ I have not looked up the statistics but are their rates of deaths of breast cancer, far lower than ours in the U.K.? If so, I am not at all surprised* R7.

This seems to be quite different to the experience in Menorca.

*We've never talked about this (having genetic testing)…. And really this is something in Menorca we are not talking about ..this problem* P3 she goes on to describe a family who were

‘*tested privately because they didn’t want to make it in Menorca because Menorca's very small, everybody knows more or less everybody*… P3.

Insights about possible national traits also include an example from USA and these could suggest that specific country appropriate, tailored interventions may need to be devised and are a salutary reminder that ‘one size does not fit all’.

‘*in the US, for example, sixty percent of young women really, really badly overestimate their risk of getting breast cancer. In the UK it’s about thirty percent of people will actually overestimate risk*' HCP1.

One of the reviewers thought the Turkey Talk detailed previously was *‘a good idea, but for us. Well, I have doubts’* R7.

### The one thing that could improve risk communication?

To conclude the RTD a specific question was asked ‘If there is one thing you think could improve risk communication; what would it be? Three of the women agreed that sending *‘everybody who has to talk to patients and their families about this on a properly accredited course’* was the answer (HCP1, P3) with the addition from one’ *that whatever the take home information is should be easy to use, easy to read, easy to understand'* P5.

On a similar information giving theme but regarding specific information or specific mechanisms of giving that information i.e. online the following comments were added,

‘*I feel very strongly that the information that’s given is so clear that people do understand that even if they have prophylactic surgery that they still can get breast cancer because I think there are a lot of people who don't know this*' P6.

And for HCP2,

*…I've learned, when a patient is experiencing a diagnosis of breast cancer or thinking about their family history of cancer what is really most useful is, in plain language, the answers to questions like, what is it like to tell my child I have breast cancer, what is it like to go for a genetic test, … for me, the future is getting some of those stories online, onto web sites, where people can click on and they can just hear a story from another patient somewhere around the world to see what is it like to…* HCP2.

The final request was for ‘*more guidance to people who are at high risk or have a predisposition, to cope with it, to bring it in their family and monitor it a little bit better*’ and to emphasize ‘*the ‘role for advocacy groups because we give conferences with neutral information*’ P4.

## Discussion

The purpose of risk communication within cancer genetics is to provide individuals with a particular family cancer history with accurate information so that they know whether they have an increased risk of cancer. It should help them to understand and interpret this risk in order to make important health care decisions and to adjust to their situation. There is a balance when addressing patient's information needs, as to what level and amount of information is required at a particular time [8] and communicators need to be able to take their cue from the recipient. Successful communication involves understanding the patient's perspective and verifying the patient's comprehension and satisfaction with the process can help ensure that the decisions reached are informed and acceptable [9]. Since the advent of patient centered care (which in today's National Health Service, UK has come to mean putting the patient and their experience at the heart of quality improvement [10]), risk communication has been considered essential to enable people to make informed decisions regarding health care. It is also recognized that this is not necessarily a decision of the moment but ‘that risk is something people live with, rather than think about and perceive at a single point in time’ and likewise decisions may need to be made at different points in time, for example regarding reproductive planning [3].

### Strengths and limitations of this research

A strength of this study is that it has asked ‘what women want’ from effective risk communication and has made video excerpts of the discussion available online for other people to view and comment and share their own experiences and examples of good practice. Its main weakness is that the women involved were, by virtue of their willingness and ability to participate in a RTD held in English probably not typical clients. However, not having women able to articulate at least some of the concerns on behalf of others would not have served anyone. Also because of the nature of the RTD (the fact that it would be filmed and hosted online) it was considered more important to ensure that the women were given oversight and felt comfortable with the proposed questions prior to the RTD than adhering to more usual practice in standard focus groups. An additional argument for our design was that the women were given opportunities to raise question or topics that they considered to be important. There were a small number of women involved in this RTD and generalizations cannot be made regarding the views discussed, but the fact that all were able to bring experiences from others in similar circumstances and that all topics discussed have at least some reference to them in the relevant literature gives added credence to their validity.

This study has given an insight into women's perspectives and their experiences of cancer genetic risk communication within their country. It has given some indication of the potential for learning and sharing what they consider to be good practice from encounters and services in different countries and has also identified the possibility of national traits that may preclude frank and open discussions that could otherwise raise awareness of genetic risk within families. Although it showed that not all women want to know their BRCA genetic status, women do want ‘skillful communicators’ who are able to identify this and have ways of describing both the possible psychosocial implications of not knowing as well as ways of communicating risks and options for those who do.

The findings presented demonstrate that there was consistency within the group around the need for improvements to the risk communication process and some of the qualities required of skillful communicators. The effectiveness of structuring the way risk is described is still being debated in the literature and women's preferences in different countries has not been sufficiently captured and documented [11]. It is clear from the RTD and the literature that information and how people have communicated with them influences their health care choices [12]. Inaccurate risk perceptions can lead to suboptimal decision making and so HCP need to be mindful of the impact their communication has on patients' understanding and subsequent beliefs and decisions based on them [13]. Perceptions of risk are affected by the way in which risk information is presented, difficulty in understanding probability and heredity and other psychological processes on the part of individuals [11,14,15].

It was suggested during the discussion that what patients ‘hear’ is not necessarily what health professionals are saying. Previous studies by d’Agincourt-Canning *et al* showed that knowledge derived from patients experience often took precedence over objective clinical estimates of risk [16]. Butow and Lobb in 2004 noted that passive listening reduces understanding especially where substantial amounts of new information are presented and that a more interactive discussion might increase understanding and recall and there are implications therefore in how information is presented and staged [17]. It was apparent from research detailed during the RTD that a patient's ability to recite verbatim information given to them does not necessarily indicate complete understanding [13].

As suggested above and also debated; how an individual interprets information is not merely based on an ability to understand the information given, but on their own belief system, personal experiences, cultural norms and an ability to cope with the possible consequences of action or inaction related to that information. The tailoring of information to take this into account becomes more complex as perhaps do the skills and strategies required to convey ‘standard’ information on the one hand, and at the same time making it unique to the individual by taking into consideration attitudes, concerns and preferences [11].

Sankar *et al* found that women decide to participate or not in BRCA1/2 genetic testing for a variety of reasons and a greater understanding of their motivation is important to ensure appropriate communication/counselling both before and after testing [18]. One of the reasons given by the women in the RTD for getting tested (or not) focused on what were seen as the opportunities or consequences of taking preventative measures to reduce breast cancer risk. Women (particularly younger women) who carry a BRCA mutation may face difficult decisions regarding risk reducing strategies such as prophylactic treatment options and these choices may have short and/or long-term consequences and significant impacts on their breast cancer risk [19].

The implications of cancer genetic testing on an individual's ability to take out insurance and possible ramifications regarding employment related issues are also an important consideration in deciding to accept or reject genetic testing [20].

Another major issue for the RTD participants was informing their families about their BRCA status; possibly not fully comprehending all of the information and implications, with what appeared to be insufficient support themselves. They were obliged to face their own ethical dilemmas and personal fear alongside a possible worry that when passed through multiple recipients the facts could be distorted and create misunderstandings and family rifts. The difficulties surrounding subsequent diffusion of risk information and interfamily communication for high-risk individuals was also identified by Hopwood who regarded communications skills training to ensure an adequate focus on the women's agenda as an area for further work [14]. Dancyger *et al* (2010) also suggest there is a value in acknowledging the motivations for genetic testing within the context of family relationships and obligations and possibly involving the wider family in counselling and decision making earlier on in cancer genetic service appointments [21].

The points above lend weight to arguments for additional materials and resources being used by health professionals to both give information and check understanding e.g. by the use of decision aids and access to online discussions such as the one described here with the opportunity to contribute and ask questions. Decision aids increase the likelihood that choices are based on realistic expectations of outcomes due to better knowledge and personal choice [22,23]. They have been shown to perform better than usual care interventions in terms of: a) greater knowledge (b) lower decisional conflict related to feeling uninformed c) lower decisional conflict related to feeling unclear about personal values d) reduced the proportion of people who were passive in decision making and e) reduced proportion of people who remained undecided post-intervention [24]. However, as Edwards *et al* discovered in their 2008 systematic review of risk communication, in interventions for both communication models and decision aids, the supportive or emotional elements of counselling provided more benefits to users than the informational or educational elements. There are other challenges associated with decision aids including how to migrate these tools to the internet to make available to a wider audience [25] particularly in the absence of appropriate supportive elements. There are potentially mechanisms to combat these challenges by incorporating decision tools within sites that are themselves interactive and could facilitate communication with health professionals and patients. This research and other studies have shown that patients would like to and benefit from speaking to others in similar circumstances [8,26,27]. Shared experiences are used for emotional and psychological support and also to acquire clinical knowledge and learn how and why other people made their decisions and manage their similar conditions. This study aims to some extent to provide this facility by making available excerpts of the RTD online and by encouraging comments or discussion. HCP are undoubtedly the first source of recognized authority but they are no longer the only ones, as patients are increasingly consulting and valuing the lived experience of other patients as a reliable source of expertise. Accessing these sources online is increasingly possible in the age of Health 2.0 via an array of websites Such as Health Talk Online (http://www.healthtalkonline.org/ which shares more than 2,000 people's experiences of over 60 health related conditions and illnesses), Cancer Genetic Story Bank (http://www.cancergeneticsstorybank.co.uk/ which shares digital stories of patients covering topics such as living with the risk of cancer and telling a family member about a cancer diagnosis) and from patient blogs and forums as described by one of the participants in the RTD. Blogs and forums can engage people in knowledge sharing and debate and can attract a large readership. They can also prompt the gathering of small virtual groupings of individuals interested in co-constructing knowledge around a common topic within a specific community. Sources such as these could potentially (alongside structured decision aids) fill both informational and emotional support requirements.

## Conclusions and recommendations

The perception of cancer risk stems not only from the way risks are stated, but also from family history, personal experiences, cultural norms and beliefs and therefore a multifaceted approach to risk communication addressing these issues is necessary to ensure the patient fully understands the potential risks. Strategies that acknowledge patient's lived experience and knowledge of the disease, may also suggest new ways to frame genetic information that will enable people to better understand their objective risk.

Women decide to participate or not in BRCA1/2 genetic testing for a variety of reasons and a greater understanding of their motivation is important for the proper counseling of women both before and after testing. A positive BRCA mutation diagnosis has implications for both patient and their family and this research indicates that a greater emphasis should be placed on supporting those communicating their status and its implications to other family members. Patients understanding and interpretation of quite complex genetic and inheritance information is often expected to be conveyed onwards within often equally complex family situations.

There is a balance when addressing patient's information needs, as to the level of detail and quantity of information that is required at a particular time. Those involved in communicating risk need to be able to take their cue from the recipient. Successful communication involves understanding the patient's perspective, verifying their understanding and ensuring the process is enabling them to reach informed decisions. Decision aids could help support the process in structuring information and options; including risks and benefits, while the ‘skilled communicator’ can confirm understanding and how this fits in with the individuals preferences. The benefits of talking to others (either in person or via online mechanisms) who have been in the same situation explaining decisions they have made and their satisfaction (or not) with those choices has not been explored as a decision aid and further research on this subject would be valuable.

Taking advantage of the potential of social media to offer patients and their families a way to enhance their learning experiences should be explored more thoroughly. Research should be conducted to determine the best ways to integrate carefully staged information, decision aids including ‘talking to people online, and also opportunities for greater online communication and collaboration between health professionals and patients, taking into account the different, but also overlapping, needs of these audiences.

## Resources

To access the video clips of the RTD please follow the following links

Information and support needs

http://www.youtube.com/watch?v=b2DQGF8m5Mw
http://bit.ly/aMAAaA

Skills of communicators

http://www.youtube.com/watch?v=PM023tU38E0&feature=player_embedded
http://bit.ly/aPiu5Z

Telling the family

http://www.youtube.com/watch?v=2gI1afNrk4U&feature=player_embedded#!
http://bit.ly/aosXkd

Improving risk communication

http://www.youtube.com/watch?v=Pp3zYGg5Elk&feature=player_embedded#!
http://bit.ly/9TthkW

To take the genetic test or not

http://ecancer.org/tv/pubdate/565
